# Epidemiological Analysis of COVID-19 Cases in Native Amazonian Communities from Peru

**DOI:** 10.3390/epidemiologia2040034

**Published:** 2021-10-09

**Authors:** Cecilia Pajuelo-Reyes, Hugo J. Valencia, Carla C. Montenegro, Eduardo Quezada, Lizandro Gonzales, Norma Cruz, Carlos Canelo, Carla Ordinola, Jorge L. Maicelo Quintana, Juan R. Tejedo, Rafael Tapia-Limonchi, Stella M. Chenet

**Affiliations:** 1 Instituto de Enfermedades Tropicales (IET), Universidad Nacional Toribio Rodríguez de Mendoza de Amazonas (UNTRM), Chachapoyas 01001, Peru; cecilia.pajuelo@untrm.edu.pe (C.P.-R.); hugo.valencia@untrm.edu.pe (H.J.V.); carla.montenegro@untrm.edu.pe (C.C.M.); carla.ordinola@untrm.edu.pe (C.O.); juan.tejedo@untrm.edu.pe (J.R.T.); rafael.tapia@untrm.edu.pe (R.T.-L.); 2 Dirección Regional de Salud (DIRESA), Chachapoyas 01001, Peru; eduquezadat2@gmail.com (E.Q.); lgonzalesc@hotmail.com (L.G.); normacruz55@hotmail.com (N.C.); 3 Gobierno Regional de Amazonas (GOREA), Chachapoyas 01001, Peru; ccanelounion@hotmail.com; 4 Instituto de Investigación para el Desarrollo Sustentable de Ceja de Selva (INDES-CES), Universidad Nacional Toribio Rodríguez de Mendoza de Amazonas (UNTRM), Chachapoyas 01001, Peru; jmaicelo@untrm.edu.pe; 5 Departamento de Biologia Molecular e Ingenieria Bioquímica, Universidad Pablo de Olavide (UPO), 41001 Sevilla, Spain; 6 Facultad de Medicina, Universidad de los Andes, Santiago de Chile 7550000, Chile

**Keywords:** native communities, COVID-19, Peru, hazard ratio, transmission chain, epidemiology, RDTs

## Abstract

Despite early control measures, SARS-CoV-2 reached all regions of Peru during the first wave of the pandemic, including native communities of the Peruvian Amazon. Here, we aimed to describe the epidemiological situation of COVID-19 in the Amazonas region of Peru using an open database of 11,124 COVID-19 cases reported from 19 March to 29 July 2020, including 3278 cases from native communities. A high-incidence area in northern Amazonas (Condorcanqui) reported a cumulative incidence of 63.84/1000 inhabitants with a much lower death rate (0.95%) than the national average. Our results showed at least eight significant factors for mortality, and the Native Amazonian ethnicity as a protective factor. Molecular confirmatory tests are necessary to better explain the high incidence of antibody response reported in these communities.

## 1. Introduction

In December 2019, a new viral respiratory disease was reported in Wuhan, China. The agent was later identified as a novel coronavirus named Severe Acute Respiratory Syndrome Coronavirus 2 (SARS-CoV-2) responsible for the Coronavirus Disease 2019 (COVID-19). It has spread worldwide as a pandemic with 85,520,927 confirmed cases and 1,849,436 deaths in 2020 [1]. Peru reported its first imported case in Lima on 6 March 2020. The patient, a 25-year-old Peruvian resident, traveled to several countries in Europe. Ten days later, on March 16th, the Government declared a national emergency, quarantine, and lockdown, and closed the borders, with only 86 confirmed cases and no deaths [2]. In 2020, the Peruvian Ministry of Health (MINSA) reported 1,017,199 confirmed cases and 37,724 deaths due to COVID-19 [3], being the eighteenth country with the highest number of cases and the thirteenth with the highest number of deaths in the world [1].

Despite early control measures, SARS-CoV-2 reached all regions of Peru, including Amazonas, which is located in the north part of the country [4]. Amazonas is divided into seven provinces: Bagua, Bongara, Chachapoyas, Condorcanqui, Luya, Rodriguez de Mendoza, and Utcubamba, with 426,806 inhabitants, representing 1.3% of the total population of Peru [5]. In 2020, Amazonas reported 27,838 confirmed cases and 408 COVID-19-related deaths [6]. The Ministry of Health strategy for COVID-19 prioritized the use of rapid diagnostic tests (RDTs) for antibody detection on suspected cases and close contacts of confirmed cases.

Condorcanqui is located in northern Amazonas, bordered by Ecuador and covered by rainforests. It is divided into three districts (Rio Santiago, El Cenepa, and Nieva) and it is home to more than 300 native communities and 42,470 inhabitants, where people live without electricity, potable water, or a sewage system, using the Santiago river as the primary means of transportation [7]. This province presents a high incidence of infectious diseases such as HIV, HBV, malaria, and other parasitic diseases. Thus, it is important to know the epidemiological situation of COVID-19 in this population which is vulnerable to syndemics with other infection diseases, including neglected tropical diseases (NTDs).

In this study, we performed an epidemiological analysis of COVID-19 cases in native populations of Amazonas at the beginning of the pandemic, from 19 March to 29 July 2020, from an open data collection of the Regional Directorate of Health of Amazonas (DIRESA).

The following sections present the results of the descriptive analysis of COVID-19 cases in Amazonas and Condorcanqui, as well as the death risk analysis of symptoms, signs, and comorbidities. Knowledge obtained from this study could be used to establish an effective surveillance and control program for COVID-19, targeting vulnerable populations such as native communities.

## 2. Materials and Methods

### 2.1. Database

**Figure 1:**
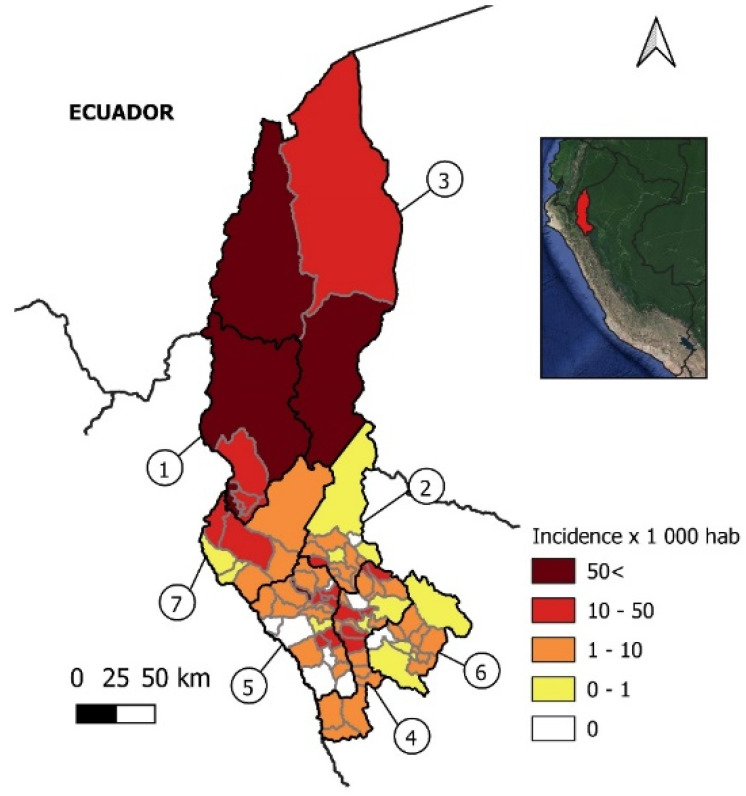
Incidence per 1000 inhabitants of COVID–19 cases in Amazonas. Political map of the Amazonas region in Peru. Provinces are within black lines and districts within gray lines. 1, Bagua; 2, Bongara; 3, Condorcanqui; 4, Chachapoyas; 5, Luya; 6, Rodriguez de Mendoza; 7, Utcubamba.

The Regional Directorate of Health of Amazonas (DIRESA) is the government agency responsible for the public health system in Amazonas, which includes COVID-19 diagnosis and epidemiological data collection. The database analyzed in this article consisted of open demographic information, clinical characteristics, and contact history of serologically confirmed cases using RDT COVID-19 IgG/IgM Rapid Test Cassette from Zhejiang Orient Gene (Biotech Co LTD, Huzhou, China). This included all cases reported from 19 March to 29 July 2020 in the seven provinces (Bagua, Condorcanqui, Chachapoyas, Utcubamba, Luya, Bongara, and Rodriguez de Mendoza) of the Amazonas region (Figure 1).

### 2.2. Variable Definitions and Descriptive Analysis

COVID-19 cases were classified as closed cases (recovered or death) and active cases. The death rate and cumulative incidence were calculated with the 2020 estimated population (426,806). Age groups were defined as children (0–11 years old), teenagers (12–17 years old), young adults (18–29 years old), adults (30–59 years old), and seniors (≥60 years old). Ethnicity included Native Amazonian of Wampis or Awajun populations, and Mestizo (people who have mixed ancestry). Case origin was defined as imported or autochthonous and confirmed positive cases with no symptoms at the moment of diagnosis were considered asymptomatic.

### 2.3. Statistical Analysis

A univariate Cox regression was used to evaluate the hazard ratio of each variable over time in symptomatic closed cases. For recovered cases, the time of recovery was calculated between the beginning of symptoms and 29 July. For death cases, the time of death was calculated between the beginning of symptoms and the date of death. Only risk factors for mortality with more than ten observations were included. After the analysis, those that fulfilled the assumption of proportionality evaluated by the log minus log plot were considered valid. Then, a multivariate Cox regression was performed to variables that fulfilled these assumptions. A fixed-effect model odds ratio (OR) with a 95% confidence interval (CI) was used to analyze the magnitude of relative risk for association between the independent variable age (all categories) in relation to the dependent variable symptomatology (asymptomatic). Statistical analyses were performed with IBM SPSS Statistics (version 21.0, SPSS Inc., Chicago, IL, USA), and graphs were made with GraphPad Prism (version 8, GraphPad Software, Inc., CA, USA).

### 2.4. Heat Maps

Maps of cumulative incidence per district in Amazonas were made and number of cases per cities in Condorcanqui province from 29 March to 29 July were monitored with QGIS Geographic Information System (version 3.10.8, QGIS Development Team, Chicago, IL, USA).

## 3. Results

### 3.1. First Reported Cases in Amazonas

**Figure 2:**
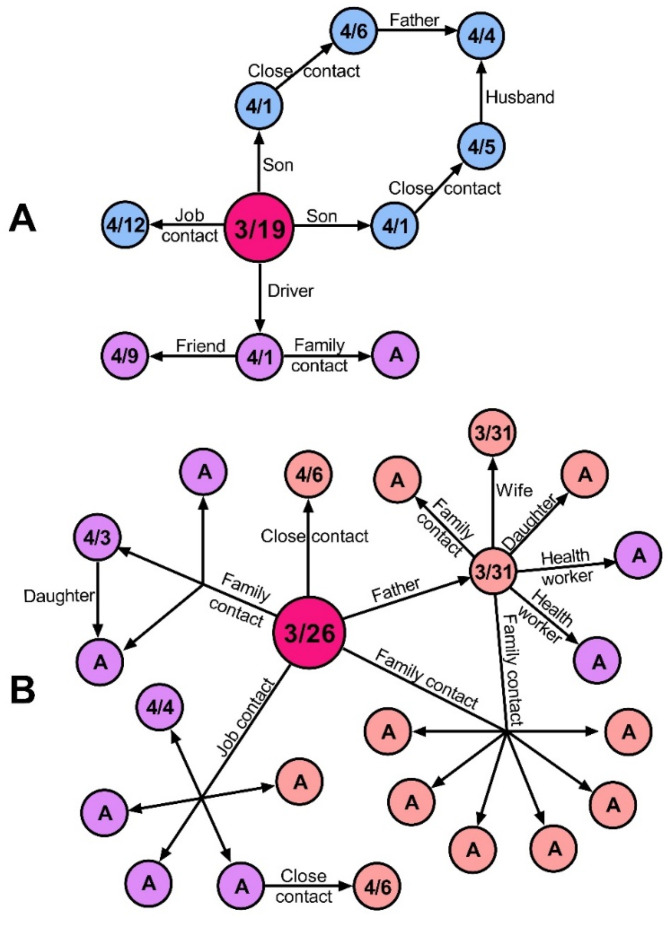
Transmission chains of the first two imported cases of COVID-19 in Amazonas, Peru. (**A**) Imported case of a policeman traveling from Lambayeque to Bagua. (**B**) Imported case of a lawyer traveling from San Martin to Bagua. Circles represent individuals. Arrows represent the direction of transmission. Sky blue, purple, and pink represent Bagua, Chachapoyas, and Bongara cases, respectively. Fuchsia color represents the primary imported case. Information in circles shows the date of symptoms onset. A, asymptomatic.

Amazonas was one of the last regions reporting COVID-19 cases in Peru. The first reported case was a policeman who traveled to Lambayeque, a region reporting a high number of COVID-19 cases at the time [8] (Figure 2a). The patient presented with fever, malaise, and cough on 26 March and was confirmed to be positive three days later by a PCR test result. His family in Bagua reported symptoms for COVID-19 on 1 April and were later confirmed to be positive by PCR on 3 April. Nine cases were confirmed after the first one, including co-workers and relatives of the policeman in Bagua and Chachapoyas. Another well-described transmission chain started with a lawyer, who traveled to San Martin Region and then returned to Bagua (Figure 2b). He presented with symptoms on 20 March but was diagnosed two weeks later, on 5 April, with a positive IgG RDT result. During those weeks he was in contact with at least 16 people, who were later confirmed to be positive, including family members, co-workers, and clients. The transmission chains of these two cases show the spread of the disease to Chachapoyas, Bagua, and Bongara.

### 3.2. Incidence of COVID-19 Cases in Amazonas

Until 29 July, Amazonas reported 11,124 confirmed cases of COVID-19 in all seven provinces, with a cumulative incidence of 26.06/1000 inhabitants. The highest incidence was found in the northern province of Condorcanqui (63.84/1000 inhabitants), followed by Bagua, Utcubamba, Chachapoyas, Luya, Bongara, and Rodriguez de Mendoza (Figure 1, Table 1). The death rate in the region was 1.92% (0.52–3.4%) with the highest values reported in Bagua (2.55%) and Utcubamba (3.4%) (Table 1).

### 3.3. Demographic Characteristics of COVID-19 Cases

From all data reported in Amazonas, including imported (3.5%) and autochthonous (96.5%) cases, 68.7% were symptomatic, with 209 associated deaths and 5 deaths with no reported symptoms. Most of the cases were adults between 30 and 59 years old, followed by young adults, seniors, teenagers, and children. Although the number of cases in men and women was similar, 76.6% of deaths occurred in male patients (Table 2).

In Condorcanqui, a total of 3278 cases were reported; 85.1% of the cases were symptomatic, mainly adults between 30 and 59 years old, farmers and housewives being the main occupations. Although the population in this province is young (21.95 ± 17.39 years old), the age group under 17 years old reported the lowest number of cases. Regarding ethnicity, 77% of the cases corresponded to Native Amazonian. The percentage of deaths was higher in males (83.9%) than in females (Table 3).

### 3.4. Clinical Characteristics

Patients in Amazonas reported the following symptoms and signs: headache (56.1%), cough (41.7%), malaise (40.9%), dyspnea or tachypnea (1%), and abnormal lung auscultation (1%). Additionally, the most frequent comorbidities were diabetes (2%) and cardiovascular diseases (1.7%). In Condorcanqui, the main signs were the same as previously mentioned, and the most frequent symptoms were headache (63.8%), malaise (59.5%), and cough (55.6%), while the comorbidities reported were diabetes (0.6%) and cardiovascular diseases (0.4%). There were 31.3% asymptomatic cases in Amazonas. The analysis of risk factors showed a significant association between people under the age of 30 and asymptomatic cases (OR = 1.689, 95% CI: 1.55–1.82, *p* < 0.001).

### 3.5. Death Risk Analysis in Closed Cases

**Figure 3:**
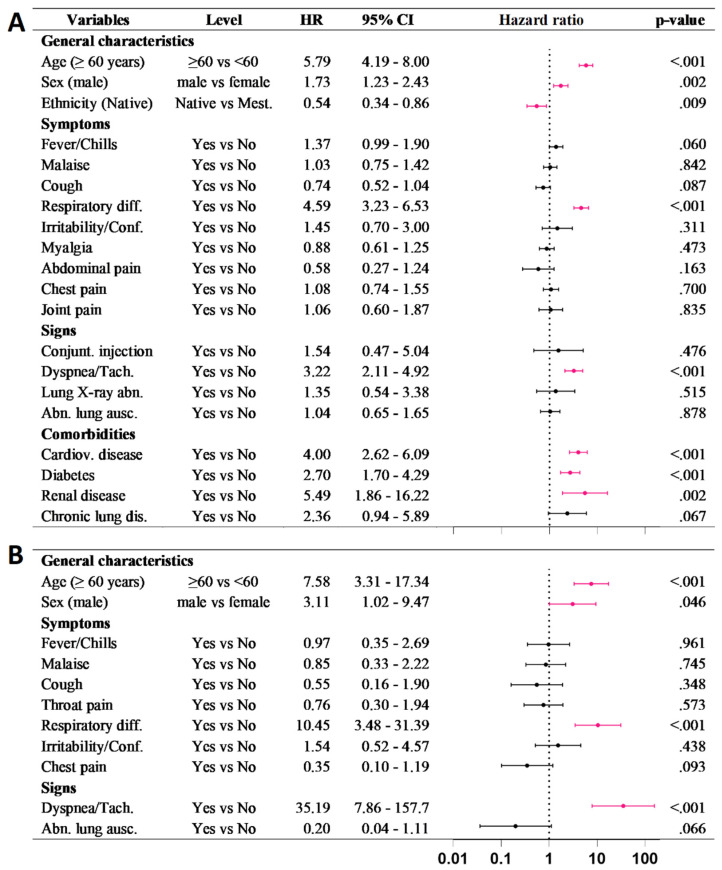
Multivariate Cox regression for death risk in closed cases of COVID–19 in (**A**) Amazonas and (**B**) Condorcanqui. Significant factors are shown in pink.

In Amazonas, the univariate Cox analysis showed 21 factors for mortality with a significant hazard ratio. Native Amazonian ethnicity was considered a protective factor (Figure S1). On the other hand, the multivariate Cox analysis identified the following significant factors associated with a higher death risk: male sex, ≥60 years, respiratory difficulty, dyspnea, diabetes, and cardiovascular and renal disease (Figure 3).

In Condorcanqui, the univariate Cox analysis showed eight factors for higher death risk with a significant hazard ratio (Figure S1). From these, the multivariate Cox analysis identified four significant factors associated with a higher death risk: male sex, ≥60 years, respiratory difficulty, and dyspnea (Figure 3).

### 3.6. Distribution and Spread of COVID-19 Cases in Condorcanqui

**Figure 4:**
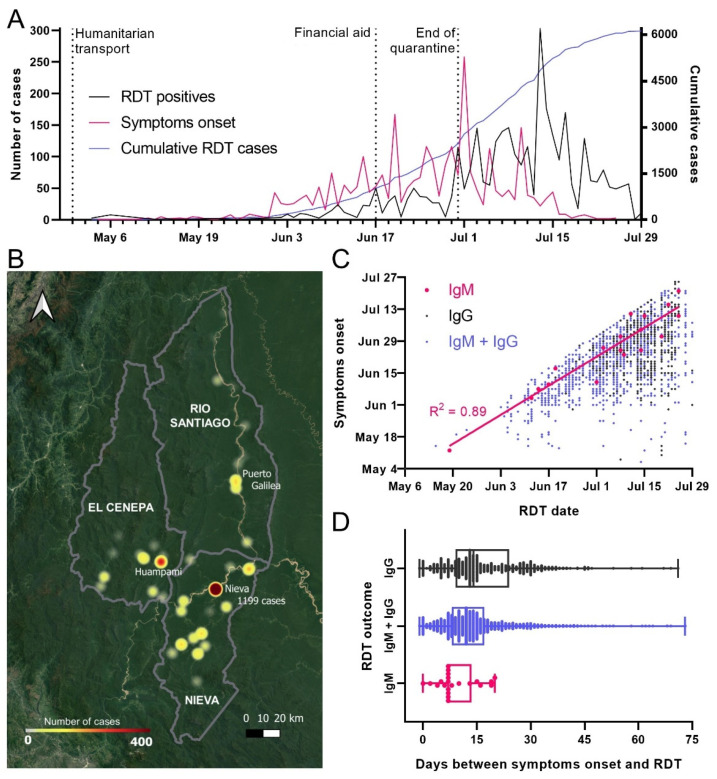
Origin and spread of COVID-19 cases in Condorcanqui. (**A**) Number of RDT-positive cases from May to July 2020 in black alongside onset of symptoms in pink. Humanitarian transport of residents back to Amazonas started in 30 April; the end of the national quarantine was on 30 June. (**B**) Cumulative number of cases of COVID-19 in localities of Condorcanqui province until 29 June. The city of Nieva and 48 local native communities from the three districts of Condorcanqui had confirmed cases of COVID-19. Nieva district had the highest incidence with the capital Santa Maria de Nieva reporting 1199 cases until 29 June. (**C**) Distribution of RDT-positive cases in Condorcanqui. Linear regression for IgM-positive cases is shown. (**D**) Box and whiskers plot of the time elapsed between the reported symptoms and the diagnosis by RDT.

Condorcanqui did not report cases until 3 May. The initial cases were reported in Nieva and corresponded to three cook assistants from Lambayeque. They were asymptomatic and diagnosed by RDT as IgG positive. By 29 July, all districts from Condorcanqui reported cases. The most populated towns were mostly affected, especially Nieva, which reported 34.1% (1119) of the cases. The distribution of cases over time showed the highest peak on July 13th corresponding to COVID-19 surveillance activities of DIRESA, with a difference of two weeks with the highest peak of onset of symptoms. A major event during that time was the financial aid given to low-income families that caused people to gather in some communities (Figure 4A,B).

Finally, according to the RDT results, IgM-only positives showed a high correlation between the date of RDT diagnosis and the onset of self-reported symptoms, with an average of 9.1 ± 5.5 days from the onset of symptoms. However, most of the cases reported both IgG + IgM (80%), followed by IgG alone (19%) (Figure 4C,D).

## 4. Discussion

There is an urgent need to understand the real situation of COVID-19 in vulnerable populations to improve surveillance and control programs. Our research represents the first epidemiological analysis of COVID-19 in native communities of Amazonas. Until 29 July 2020, the cumulative incidence per 1000 inhabitants in Condorcanqui (63.84) was higher than the one reported in Amazonas (26.06), Peru (12.49), and worldwide (2.24) [9,10], showing the importance of studies in this province.

Even though Amazonas was one of the last regions of Peru to report COVID-19 cases, these rapidly expanded to the northern provinces of Bagua and Condorcanqui, where Amazonian native communities live. The major reasons for the disease expansion were: insufficient SARS-CoV-2 testing, lack of compliance with the contingency measures (travel restriction, lockdown, and social distancing), and the agglomeration of the population in major cities and villages to obtain government financial aid. Additionally, in Condorcanqui, only 7.82% of the population has access to drinking water [7], which reduces hygiene measures while their habits include self-medication and the use of traditional medicine. Moreover, weather conditions and hard labor jobs make the use of masks and other PPEs in these communities difficult. Finally, other important factors are the shortage of health care personnel and difficulty in accessing the area, which could result in late intervention, diagnosis, and treatment. Besides, the presence of neglected tropical diseases such as dengue, chikungunya, leishmania, and other infectious diseases such as HIV, tuberculosis, endemic malaria vivax, and a recent *Plasmodium falciparum* outbreak [11,12] raises the possibility of syndemics and further complications with COVID-19 [13,14]. It is worth noting that although the number of cases increased rapidly in Amazonas and the cumulative incidence in Condorcanqui was higher than in other provinces, the death rate was lower (0.95%) compared to Bagua (2.55%) and Utcubamba (3.4%). Moreover, the death rate reported for 2020 in Condorcanqui (0.65%) was lower than the average death rate in Amazonas (1.47%) and Peru (3.7%) [3,6].

Overall, our analysis showed that risk factors for mortality in COVID-19 cases from Amazonas such as male sex, age (>60), respiratory distress, dyspnea, diabetes, and cardiovascular and renal disease were similar to those previously reported [15,16,17,18,19,20]. On the other hand, we found that ethnicity could be considered a protective factor. The reason why Native Amazonians do not present further complications that lead them to death is not clear and it is in contrast with the high incidence reported in Condorcanqui. Possible reasons include the young age structure of the population and their low-calorie diet, without processed foods, which explains the low prevalence of comorbidities such as diabetes and cardiovascular diseases. Moreover, intrinsic immunological factors could be playing a major effect because of the high incidence of infectious diseases. For instance, chronic intestinal parasitic diseases could be related to an immunosuppressive response and possible mitigation of COVID-19 morbidity and mortality [13,21]. Furthermore, antibodies anti-glycosylphosphadylinositol (GPI), developed in malaria infection, could cross react with glycoproteins (GPs), playing a protective role against COVID-19 [22]. Additionally, the frequency of human leukocyte antigen (HLA) alleles and microbiota in these isolated populations could influence the immune response against COVID-19 [23,24,25,26].

On the other hand, several key considerations about the diagnosis limit the interpretation of our findings, such as the exact symptom onset dates reported by the participants, and the use of RDTs with lower sensitivity and specificity [27] compared to molecular testing. These limitations might underestimate or overestimate the true proportion of infected people. Additionally, since the presence of asymptomatic infections could contribute to the spreading of SARS-CoV-2, screening the population is necessary to establish the true transmission dynamics of the infections and improve control measures [28].

Although previous serological studies in Peru have reported values of 20.8% in Lima with equal distribution across all age groups [29] and 60% in Iquitos with children under 12 being the most vulnerable to this disease [30], our results need to be interpreted with caution. First of all, we used data collected by the DIRESA which initially directed the diagnosis towards the population with symptoms; therefore, prevalence values cannot be directly compared to the ones obtained from the previous serological studies. Second, performance of RDTs assays are not completely reliable since reactivity could vary geographically and might be in part related to previous human coronavirus exposure or infections by other pathogens [31,32,33,34]. This could also explain the high cumulative rate incidence reported in Condorcanqui, with false positives cross reacting with antibodies from other infectious diseases such as malaria [33]. Therefore, further validations and follow-up studies are needed to determine the true population affected by SARS-Cov-2, especially in native communities.

## 5. Conclusions

This represents the baseline study of the epidemiological situation of Native Amazonian communities in Condorcanqui. We reported a high COVID-19 incidence and a low death rate in these communities compared to the rest of the region and even to the rest of the country, with common risk factors. Additionally, Native Amazonian ethnicity was reported as a protective factor. However, we emphasize that follow-up studies and molecular tests are needed to determine the real impact of COVID-19 in native communities.

Moving forward, we propose a change in the surveillance strategy of COVID-19 cases in native communities of Condorcanqui and a more extended epidemiological study that revises unreported deaths. For example, it would be valuable to include the use of molecular tests with minimum laboratory equipment for non-accessible areas. Moreover, it is important to consider that the epidemiological questionnaire for COVID-19 in Peru is standard for all populations and some of the questions might not be applicable for these native communities and should include key factors such as infectious diseases circulating in the area or self-medication. Studies about the effects in the health system and syndemics with malaria and HIV will be crucial for understanding the real impact of the pandemic in native communities and preventing future outbreaks and deaths. Knowledge of the characteristics and risk factors associated with this disease provides a starting point to establish an efficient regional program to target vulnerable groups and perform an efficient vaccination campaign. This program should be a multidisciplinary effort to understand the disease and the behavior of the communities and to actively engage the population in preventive measures.

## Figures and Tables

**Table 1:** COVID-19 confirmed cases in the seven provinces of Amazonas Region until 29 July.

Province	Population	Active Cases	Recovered Cases	Deaths	Death Rate	Cumulative Incidence × 1000 hab.
Bagua	84,672	3550	573	108	2.55%	49.97
Bongará	26,830	170	32	0	-	7.53
Condorcanqui	51,344	2359	888	31	0.95%	63.84
Chachapoyas	63,188	567	197	4	0.52%	12.15
Luya	47,827	307	189	4	0.80%	10.45
Rodríguez de Mendoza	33,651	111	62	0	-	5.14
Utcubamba	119,294	1420	485	67	3.40%	16.53
Total in Amazonas	426,806	8484	2426	214	1.92%	26.06

**Table 2:** Characteristics of COVID-19 confirmed cases in Amazonas region.

	Total		Active Cases		Recovered Cases		Deaths	
	*N*	%	*N*	%	*N*	%	*N*	%
Age group								
Children (0–11)	533	4.8%	419	3.3%	113	4.7%	1	0.5%
Teenagers (12–17)	513	4.6%	402	3.9%	111	4.6%	0	0%
Young Adults (18–29)	2740	24.6%	2110	24.6%	624	25.7%	6	2.8%
Adults (30–59)	6024	54.2%	4615	58.6%	1346	55.5%	63	29.4%
Seniors (≥60)	1314	11.8%	938	9.6%	232	9.6%	144	67.3%
Gender								
Male	5569	49.9%	4111	43.7%	1294	53.3%	164	76.6%
Female	5555	50.1%	4373	56.3%	1132	46.7%	50	23.4%
Ethnicity								
Native Amazonian	3919	35.2%	3197	37.7%	694	28.6%	28	13.1%
Mestizo	7204	64.8%	5286	62.3%	1732	71.4%	186	86.9%
Origin								
Imported	392	3.5%	195	2.3%	193	8.0%	4	1.9%
Autochthonous	10,732	96.5%	8289	97.7%	2233	92.0%	210	98.1%
Occupation								
Housewife	3099	28%	2612	31%	452	19%	35	16%
Farmer	1809	16%	1459	17%	315	13%	35	16%
Teacher	664	6%	509	10%	139	6%	16	7%
Health care	675	6%	374	4%	295	12%	6	3%
Drivers	266	2%	195	2%	58	2%	13	6%
Police and military	602	5%	405	5%	182	8%	15	7%
Merchants	527	5%	355	4%	159	7%	13	6%
Student	1108	10%	885	10%	222	9%	1	0%
Other	1810	16%	1276	15%	478	20%	56	27%
Not active	564	5%	414	5%	126	5%	24	11%
Total	11,124	100%	8484	100%	2426	100%	214	100%

**Table 3:** Characteristics of COVID-19 confirmed cases in Condorcanqui province.

	Total		Active Cases		Recovered Cases		Deaths	
	*N*	%	*N*	%	*N*	%	*N*	%
Age group								
Children (0–11)	99	3%	77	3.3%	22	2.5%	0	0%
Teenagers (12–17)	116	3.5%	92	3.9%	24	2.7%	0	0%
Young Adults (18–29)	830	25.3%	580	24.6%	248	27.9%	2	6.5%
Adults (30–59)	1925	58.7%	1383	58.6%	529	59.6%	13	41.9%
Seniors (≥60)	308	9.4%	227	9.6%	65	7.3%	16	51.6%
Gender								
Male	1557	47.5%	1032	43.7%	499	56.2%	26	83.9%
Female	1721	52.5%	1327	56.3%	389	43.8%	5	16.1%
Ethnicity								
Native Amazonian	2523	77%	1873	79.4%	631	71.1%	19	61.3%
Mestizo	755	23%	486	20.6%	257	28.9%	12	38.7%
District								
Nieva	2237	68%	1624	69%	595	67%	18	58%
El Cenepa	668	20%	471	20%	191	22%	6	19%
Rio Santiago	373	11%	264	11%	102	11%	7	23%
Origin								
Imported	41	1%	1	0%	39	4.4%	1	3.2%
Autochthonous	3237	99%	2358	100%	849	95.6%	30	96.8%
Occupation								
Housewife	1199	36.6%	998	42.3%	196	22.1%	5	16.1%
Farmer	868	26.5%	649	27.5%	206	23.2%	13	41.9%
Teacher	206	8.3%	177	7.5%	91	10.2%	5	16.1%
Health care	188	6.3%	59	2.5%	145	16.3%	2	6.5%
Student	51	5.7%	140	5.9%	47	5.3%	1	3.2%
Other	239	9.3%	109	4.6%	128	8.4%	2	6.5%
Not active	305	10.1%	227	9.6%	75	14.4%	3	9.7%
Total	3278	100.0%	2359	100.0%	888	100.0%	31	100.0%

## Data Availability

The data presented in this study are available on https://app.powerbi.com/view?r=eyJrIjoiMDM4OTdiMzktYWQyOC00MWQ0LTk3NDctZWRjY2VhM2MxOWFmIiwidCI6IjM0MGJjMDE2LWM2YTYtNDI2Ni05NGVjLWE3NDY0YmY5ZWM3MCIsImMiOjR9 (accessed on 25 September 2021) and https://www.dge.gob.pe/portalnuevo/covid-19/covid-cajas/situacion-del-covid-19-en-el-peru/ (accessed on 25 September 2021).

## Supplementary Materials

The following are available online at https://www.mdpi.com/article/10.3390/epidemiologia2040034/s1, Figure S1: Univariate Cox regression for death risk in closed cases of COVID-19 in (**A**) Amazonas and (**B**) Condorcanqui. Significant factors are shown in pink.
