# Supplementary material for: Epidemiological Analysis of COVID-19 Cases in Native Amazonian Communities from Peru
Source: Epidemiologia (Basel). 2021 Oct 9;2(4):490–501. doi: 10.3390/epidemiologia2040034 (PMC9620947; doi:10.3390/epidemiologia2040034)
Supplement: Supplementary file 1 [file epidemiologia-02-00034-s001.zip › epidemiologia-1383725-supplementary.pdf]

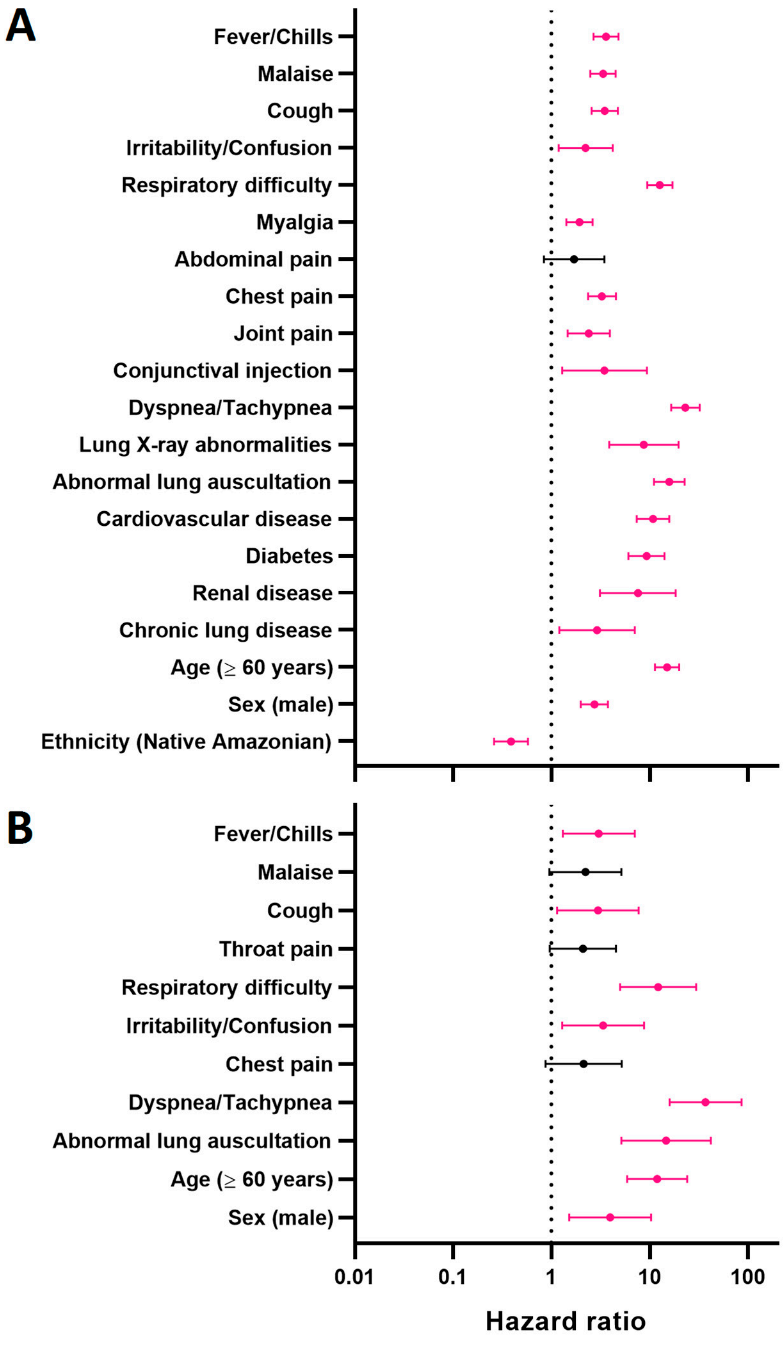

Figure S1. Univariate Cox regression for death risk in closed cases of COVID-19 in (**A**) Amazonas and (**B**) Condorcanqui. Significant factors are shown in pink.
